# Rivaroxaban as Secondary Thromboprophylaxis in Pediatric Patients With Short Bowel Syndrome

**DOI:** 10.1155/crh/7383741

**Published:** 2026-09-29

**Authors:** Angela F. Journell, Chelsea E. Peters, Jessica Meznarich, Sasidhar Goteti

**Affiliations:** ^1^ Intermountain Health, Primary Children’s Hospital, Salt Lake City, Utah, USA; ^2^ Department of Pediatrics, Division of Pediatric Hematology/Oncology, University of Utah, Salt Lake City, Utah, USA, utah.edu

**Keywords:** digestive malabsorption, rivaroxaban, thromboprophylaxis

## Abstract

Pediatric patients with short bowel syndrome often require long‐term parenteral nutrition via a central venous catheter (CVC), which places them at risk for venous thromboembolism (VTE). Our objective was to retrospectively review a single‐center use of rivaroxaban in patients with short bowel syndrome (*n* = 3) to determine if rivaroxaban is viable secondary thromboprophylaxis. One out of three patients developed a new VTE while their prophylactic rivaroxaban was held, one patient developed a gastrointestinal bleed, and one patient completed therapy without an event. Rivaroxaban may be feasible in selected patients with short bowel syndrome with close therapeutic drug level monitoring, but larger studies are needed.

## 1. Introduction

Pediatric venous thromboembolism (VTE) occurs in 0.07–0.49 per 10,000 children with notably higher rates in hospitalized children, 4.9–21.9 per 10,000 [1, 2]. The risk of VTE is significantly increased in patients who require central venous catheters (CVCs), with up to 85% of pediatric VTE being associated with a CVC [3]. Various conditions that lead to digestive malabsorption may require long‐term CVCs for parenteral nutrition, and patients with intestinal failure who require long‐term parenteral nutrition are at increased risk of VTE [4–7]. VTE is associated with significant morbidity and mortality including increased hospital length of stay, recurrent VTE, and post‐thrombotic syndrome. Development of a VTE may lead to permanent loss of line access in patients who require CVC placement for life‐sustaining parenteral nutrition, and prophylactic anticoagulation can reduce VTE risk [8, 9]. Low‐molecular‐weight heparin (LMWH), or enoxaparin, has been the standard of care for pediatric patients requiring VTE prophylaxis [10]. For patients requiring chronic anticoagulation, LMWH is often not preferred by patients or their caregivers since it is administered as a subcutaneous injection once or twice a day. Chronic administration of LMWH can lead to medical trauma and poor compliance in this patient population, and they may favor an oral option [11]. Vitamin K antagonists, an alternative anticoagulation agent, are poorly favored due to many drug and food interactions and the need for frequent laboratory monitoring [12]. In December 2021, the direct oral anticoagulant (DOAC) rivaroxaban was approved for pediatric use for treatment of acute VTE [13] and prophylaxis in post‐Fontan pediatric patients [14]. Rivaroxaban is a selective and reversible factor Xa inhibitor and is primarily absorbed in the stomach with limited absorption distal to the stomach [15]. There is a gap in research on the use of rivaroxaban in pediatric patients, specifically in patients with digestive malabsorptive conditions like short bowel syndrome. We describe our experience with trialing rivaroxaban for secondary VTE prophylaxis in 3 patients with a history of CVC‐associated VTE who require chronic total parenteral nutrition (TPN) due to a known short bowel syndrome at our institution.

## 2. Methods

### 2.1. Data Extraction

We retrospectively reviewed the electronic medical record of patients < 18 years of age with short bowel syndrome who required anticoagulation to prevent VTE between 2022 and 2024. These patients were trialed on rivaroxaban with measurement of rivaroxaban peak levels drawn 2–4 h after dosing. The rivaroxaban peak levels were processed at our local ARUP laboratory using a chromogenic antifactor Xa assay. Ranges for DOAC drug levels have not been established; instead, we used rivaroxaban plasma concentration levels from patient populations studied in adult data for VTE prevention after total hip replacement surgery following a 10‐mg once daily dose (C‐max postdose peak plasma concentration 91–196 ng/mL) [16]. Rivaroxaban doses were titrated to achieve the expected prophylactic range. Outcomes are described including major bleeding, clinically relevant nonmajor bleeding or clotting events, and future anticoagulation treatment.

### 2.2. Ethics Approval

This study was approved by the Institutional Review Boards at the University of Utah and Primary Children’s Hospital, Salt Lake City, UT, United States (IRB_00188583). Informed consent was waived due to the retrospective nature of the study and was approved under the institutional IRB.

### 2.3. Cases

Case 1: 22‐month‐old female patient with a history of TPN dependence due to short bowel syndrome–associated intestinal failure (SBS‐associated IF) with a tunneled Broviac in place and a history of a CVC‐associated VTE treated with LMWH. Her short‐bowel anatomy is duodeno–jejunal, ileo–ileo, and ileo–colonic (transverse) anastomoses with at least 12% of expected bowel length remaining, with an estimated 25 cm of small bowel remaining. Her ileocecal valve is absent; bowels are in continuity with partial colon present. It was determined that she would benefit from secondary thromboprophylaxis. The parents expressed concern that she would not tolerate LMWH injections, and with shared decision‐making, she was started on prophylactic rivaroxaban at a starting weight‐based dose using the pediatric dose recommendation for thromboprophylaxis after a Fontan procedure. She had rivaroxaban peak levels drawn every 1–2 months with a range of 27–134 ng/mL (expected prophylactic range 91–196 ng/mL), and her dose was escalated from 1.7 mg twice daily up to 4 mg twice daily (Figure 1) due to peak rivaroxaban levels below the expected prophylactic range (Supporting Table 1a). She had serial ultrasounds every 6 months to assess for CVC‐associated VTE recurrence; all imaging was negative with resolution of the previous CVC‐associated VTE. Her CVC was removed at age 3 and her anticoagulation was discontinued (Table 1).

**FIGURE 1:**
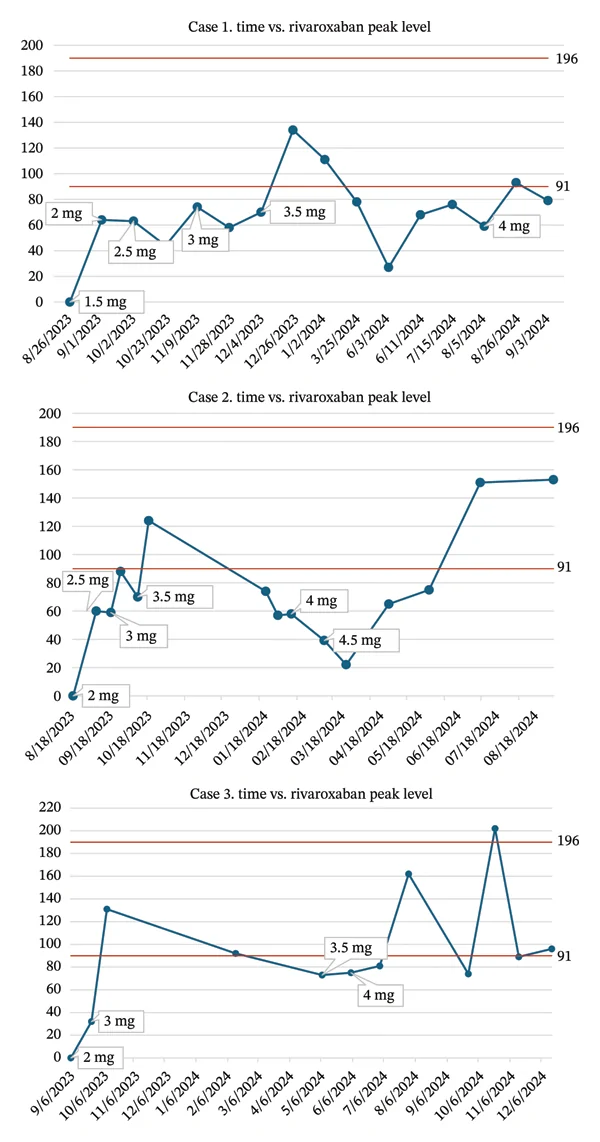
Initial and subsequent rivaroxaban peak levels in 3 patients with SBS requiring secondary thromboprophylaxis.

**TABLE 1:** Patient characteristics and outcomes.

	Case 1	Case 2	Case 3
Age at rivaroxaban thromboprophylaxis initiation	22 months	5 years	4 years
Sex	F	F	M
Weight at rivaroxaban thromboprophylaxis initiation (kg)	10	17.6	14.7
Estimated functional gut due to short bowel syndrome	12%	53%	16%
Estimated small bowel remaining (cm)	25	100	40
Ileocecal valve	Absent	Absent	Absent
Bowel continuity	Y	Y	Y
CVC	Y	Y	Y
Length of parenteral anticoagulation prior to rivaroxaban prophylaxis	None	2 years	2 years
Expected prophylactic range (ng/mL)	91–196	91–196	91–196
Peak level range (ng/mL)	27–134	22–151	32–162
Number of peak levels obtained	16	15	12
Duration of rivaroxaban exposure	12 months	13 months	15 months
Outcome	CVC removed	New VTE^∗^	GI bleed
Current anticoagulant	No longer on anticoagulation	Switch to LMWH	No longer on anticoagulation

^∗^Prophylactic rivaroxaban was held during hospitalization for CVC infection; patients developed CVC‐associated VTE.

Case 2: 5‐year‐old female patient with TPN dependence due to SBS‐associated IF with a tunneled Broviac in place. Her short bowel anatomy is jejuno–colonic anastomosis (jejuno–rectal) with at least 53% of expected bowel length remaining, with an estimated 100 cm of small bowel remaining. Her ileocecal valve is absent; bowels are in continuity with partial colon present. She developed a CVC‐associated VTE and received therapeutic LMWH for 8 weeks, at which time there was interval resolution of thrombus, so LMWH was discontinued. Immediately after discontinuation of LMWH, she developed a new CVC‐associated VTE, which was treated with therapeutic LMWH for another 3 months. After completion of her VTE treatment, she was transitioned to prophylactic LMWH but had difficulty maintaining adequate anti‐Xa levels due to noncompliance. She was transitioned to prophylactic rivaroxaban due to discomfort with LMWH injections, noncompliance, and difficulty with anti‐Xa laboratory monitoring. She had rivaroxaban peak levels drawn every 1–2 months with a range of 22–151 ng/mL (expected prophylactic range 91–196 ng/mL), requiring multiple dose escalations from 2 mg twice daily to 4.5 mg twice daily (Figure 1 and Supporting Table 1b). At age 7, she was admitted for a CVC line infection, and her rivaroxaban was held for approximately 7 days for thrombocytopenia and anticipated line removal. During this time, she was diagnosed with a VTE associated with her left IJ Broviac catheter prior to removal. The thrombus was still present on repeat ultrasound after the CVC was removed. Her anticoagulation was then transitioned to therapeutic LMWH (Table 1).

Case 3: 4‐year‐old male patient with TPN dependence due to SBS‐associated IF with a tunneled Broviac in place and a history of nonocclusive CVC‐associated VTE treated with LMWH. His short bowel anatomy is a jejuno–colonic anastomosis with at least 16% of expected bowel length remaining, with an estimated 40 cm of small bowel remaining. His ileocecal valve is absent; bowels are in continuity with partial colon present. Due to the chronic need for CVC, he had continued LMWH at prophylactic dosing for secondary thromboprophylaxis for approximately 2 years; however, due to concerns about intolerance of twice‐daily LMWH injections, with shared decision‐making, he was transitioned to prophylactic rivaroxaban. His starting dose was weight‐based using the pediatric dose recommendation for thromboprophylaxis after a Fontan procedure. He had rivaroxaban peak levels drawn monthly with a range of 32–162 ng/mL (expected prophylactic range 91–196 ng/mL); his dose was escalated from 2 mg twice daily to 4 mg twice daily (Figure 1) to achieve consistent peak levels within the expected prophylactic range (Supporting Table 1c). At age 6, anticoagulation was stopped due to bleeding concerns, with GI ulcers found on colonoscopy that were thought to be anastomotic ulcers, but rivaroxaban was held given positive fecal occult and a 2 g/dL decrease in hemoglobin. He did not require supplemental PRBC transfusion but did receive IV iron supplementation. At the time of this bleeding event, his most recent rivaroxaban peak level was 96 ng/mL. He has remained off anticoagulation for 4 months, with no clinical signs of recurrent VTE (Table 1).

## 3. Results and Discussion

Adequate treatment and prevention of VTE with anticoagulation are important for preservation of vascular access and avoidance of complications like loss of CVC, prolonged hospitalizations, and post‐thrombotic syndrome. Anticoagulant options are limited for pediatric patients with the potential for poor compliance with the standard of care options, LMWH injections, and warfarin. However, the recent FDA approval of rivaroxaban for treatment of VTE in pediatric patients based on data from the EINSTEIN Jr trial provides new opportunities to assess its use in special populations, such as those with digestive malabsorption [13, 17]. There is evidence of limited DOAC absorption in patients who have undergone gastrointestinal tract surgery [18]. Since there is concern for insufficient rivaroxaban levels in this patient population due to variability in intestinal anatomy and absorption, it is reasonable to consider drug level monitoring and dose titration to ensure that rivaroxaban levels are within the expected prophylactic range while also recognizing that there is potential for interpatient and intrapatient variability in DOAC drug levels, which may limit interpretability [19].

Our case series summarizes three pediatric patients with short bowel syndrome who were treated with rivaroxaban and utilized rivaroxaban peak levels to ensure that the expected prophylactic ranges were achieved. We utilized the weight‐based pediatric dose recommendation for thromboprophylaxis after a Fontan procedure [14]. It should be noted that all three patients required dose escalations, so utilizing the recommended dose for thromboprophylaxis after a Fontan procedure may be inappropriate in patients with short bowel syndrome [14]. In our experience, two patients with short bowel syndrome and a chronic CVC were treated with rivaroxaban for secondary thromboprophylaxis for > 1 year at varying doses without recurrent VTE or major bleeding events. It is important to highlight that the patient in Case 2 redeveloped a VTE when prophylactic rivaroxaban was held for 7 days in the setting of CVC infection with CVC replacement. One patient discontinued rivaroxaban due to clinically relevant nonmajor bleeding; however, the primary source of bleeding was felt to be secondary to anastomotic ulcers, not his rivaroxaban. Outcomes from this case series suggest that rivaroxaban is potentially an appropriate anticoagulation option in patients with digestive malabsorption due to SBS‐associated IF. These three patients with SBS‐associated IF did have normal weight gain throughout this study and required multiple rivaroxaban dose adjustments with frequent rivaroxaban peak level monitoring. We increased each patient’s rivaroxaban dose based on consistently low rivaroxaban peak level results despite parental report of appropriate rivaroxaban dose administration (for example, no recent missed rivaroxaban dose and rivaroxaban dose was administered with food). The peak levels varied significantly, which may be attributed to variability in the intestinal length of each patient, making it difficult to draw a conclusion in this specific patient population.

In a review of the literature, there is a similar case report on the use of rivaroxaban for VTE treatment and secondary thromboprophylaxis in a pediatric patient with SBS and congenital nephrotic syndrome; this patient also required titration of their rivaroxaban dose to reach adequate peak levels [20]. There is a case series supporting the use of rivaroxaban in three adult patients with SBS, which found adequate peak rivaroxaban levels in all three adult patients on standard rivaroxaban dosing of 20 mg daily [21]. The PDER PAN study evaluated the pharmacokinetics of the DOACs dabigatran and rivaroxaban in six adult patients with SBS; there was evidence of DOAC absorption, although their plasma drug levels were lower than reference values, and there was significant interindividual variability [22]. After completion of our case series, Hilberath et al. published a case series of rivaroxaban use as secondary thromboprophylaxis in 26 pediatric patients with intestinal failure, including SBS. Most patients required rivaroxaban dose titration to achieve rivaroxaban trough and peak levels within the expected treatment range, but the description of dose titration was not reported. In terms of adverse events, 2/26 patients (7.7%) experienced noninfectious catheter‐related thrombus, and 4/26 patients (15%) experienced an adverse bleeding event described as GI bleeding [23]. Our study differs from Hilberath et al. in that we were targeting rivaroxaban peak levels within the expected prophylactic range. Our thrombosis event was in the setting of central line–related infection; however, like Hilberath et al., our adverse bleeding event was a GI bleed.

There are several limitations to our study including a small sample size and a retrospective nature. Furthermore, other institutions may not have easy access to therapeutic rivaroxaban level monitoring, affecting prompt turnaround time and treatment management. Ranges for DOAC drug levels have not been established; instead, we targeted peak levels within the expected prophylactic range derived from clinical trials evaluating the use of DOACs in adults with atrial fibrillation and VTE [16]. Our institution did not trial alternative DOACs, such as apixaban or dabigatran. In addition, there is substantial interpatient and intrapatient variability in DOAC drug levels, which limits their interpretation [19]. Further research is needed to establish the optimal starting dose and target peak levels for DOACs in pediatric patients with short bowel syndrome and digestive malabsorption.

## Author Contributions

Angela F. Journell analyzed data and wrote the manuscript. Chelsea E. Peters analyzed data and wrote the manuscript. Jessica Meznarich analyzed data and wrote the manuscript. Sasidhar Goteti designed the study, analyzed data, and wrote the manuscript.

## Funding

No funding was acquired for this study.

## Disclosure

All authors have read and approved the final version of the manuscript. Sasidhar Goteti had full access to all of the data in this study and takes complete responsibility for the integrity of the data and the accuracy of the data analysis.

## Conflicts of Interest

The authors declare no conflicts of interest.

## Supporting Information

Additional supporting information can be found online in the Supporting Information section.

## Supporting information


**Supporting Information 1** Supporting Table 1a. Dosage changes with corresponding levels and comments for Case 1. Supporting Table 1b. Dosage changes with corresponding levels and comments for Case 2. Supporting Table 1c. Dosage changes with corresponding levels and comments for Case 3.

**Supporting Information 2** DOAC CARE checklist Goteti.

## Data Availability

The data that support the findings of this study are available from the corresponding author upon reasonable request.
