# Supplementary material for: Rivaroxaban as Secondary Thromboprophylaxis in Pediatric Patients With Short Bowel Syndrome
Source: Case Rep Hematol. 2026 Sep 29;2026:7383741. doi: 10.1155/crh/7383741 (PMC13621405; doi:10.1155/crh/7383741)
Supplement: Supplementary file 1 [file CRH-2026-7383741-s001.docx]

| Date | Dose | Level | Dose change | Comment |
| --- | --- | --- | --- | --- |
| 8/27/23 | 1.7 mg BID |  |  |  |
| 9/1/23 | 1.7 mg BID | 64 ng/mL | 2 mg BID | Increase dose, check in 1 week |
| 10/2/23 | 2 mg BID | 63 ng/mL | 2.5 mg BID | Increase dose, check in 1 week |
| 10/23/23 | 2.5 mg BID | 44 ng/mL | none | GI illness with vomiting, rivaroxaban was held during this GI illness |
| 11/9/23 | 2.5 mg BID | 74 ng/mL | 3 mg BID | Still having vomiting but increased dose, check in 1 week |
| 11/28/23 | 3 mg BID | 58 ng/mL | none | Missed a dose the week prior, check in 1 week |
| 12/4/23 | 3 mg BID | 70 ng/mL | 3.5 mg BID | Increase dose, check in 1 week |
| 12/26/23 | 3.5 mg BID | 134 ng/mL | none | Check in 3 months |
| 3/25/24 | 3.5 mg BID | 78 ng/mL | none | Recent change of GI medication (stopped loperamide prior to endoscopy), continued 3.5 mg BID after restarting loperamide, check in 1 week |
| 6/3/24 | 3.5 mg BID | 27 ng/mL | none | Experienced multiple delays with obtaining a repeat rivaroxaban peak level. No missed doses or recent medication changes, will repeat in a few days |
| 6/11/24 | 3.5 mg BID | 68 ng/mL | none | Check in 1 month |
| 7/15/24 | 3.5 mg BID | 76 ng/mL | none | Check in 1 month |
| 8/5/24 | 3.5 mg BID | 59 ng/mL | 4 mg BID | Increase dose, check in 1-2 weeks |
| 8/22/24 | 4 mg BID | 93 ng/mL | none | Check in 1 week |
| 9/3/24 | 4 mg BID | 79 ng/mL | none | Check in 1 month |

Supplemental Table 1a. Dosage changes with corresponding levels and comments for Case 1

| Date | Dose | Level | Dose change | Comment |
| --- | --- | --- | --- | --- |
| 8/27/23 | 2 mg BID |  |  |  |
| 9/6/23 | 2 mg BID | 60 ng/mL | 2.5 mg BID | Increase dose, check in 1 week |
| 9/18/23 | 2.5 mg BID | 59 ng/mL | 3 mg BID | Increase dose, check in 1 week |
| 9/26/23 | 3 mg BID | 88 ng/mL | 3.5 mg BID | Increase dose, check in 1 week |
| 10/9/23 | 3 mg BID | 70 ng/mL | 3.5 mg BID | Missed doses and was only giving 3 mg so increased to 3.5 mg, check in 1 week |
| 10/19/23 | 3.5 mg BID | 124 ng/mL | none | Check in 3 months |
| 1/23/24 | 3.5 mg BID | 74 ng/mL | none | Delayed result, then patient was admitted |
| 2/2/24 | 3.5 mg BID | 57 ng/mL | 4 mg BID | Drawn during admission. Increase dose, check in 1 week |
| 2/13/24 | 4 mg BID | 58 ng/mL | 5 mg BID | Increase dose, check in 1 week |
| 3/11/24 | 4 mg BID | 39 ng/mL | 4.5 mg BID | Was only giving 4 mg and level drawn >4 hours after dose. Increase dose, check in 1 week |
| 3/29/24 | 4.5 mg BID | 22 ng/mL | none | Discussed switching back to enoxaparin, but mom hesitant and reports recent change of GI medication (increased teduglutide dose), mom opted to continue current dose and check in 1 month |
| 5/3/24 | 4.5 mg BID | 65 ng/mL | none | Check in 1 month |
| 6/5/24 | 4.5 mg BID | 75 ng/mL | none | Check in 1 month |
| 7/17/24 | 4.5 mg BID | 151 ng/mL | none | Check in 1 month |
| 9/15/24 | 4.5 mg BID | 153 ng/mL | None | Check in 3 months |

Supplemental Table 1b. Dosage changes with corresponding levels and comments for Case 2

| Date | Dose | Level | Dose change | Comment |
| --- | --- | --- | --- | --- |
| 9/13/23 | 2 mg BID |  |  |  |
| 9/26/23 | 2 mg BID | 32 ng/mL | 3 mg BID | Increase dose, check in 1 week |
| 10/11/23 | 3 mg BID | 131 ng/mL | 3 mg BID | Check in 3 months or when weight >20 kg |
| 2/13/24 | 3 mg BID | 92 ng/mL | none | Check in 3 months |
| 5/7/24 | 3 mg BID | 73 ng/mL | 3.5 mg BID | Increase dose, check in 1 week |
| 6/4/24 | 3.5 mg BID | 75 ng/mL | 4 mg BID | Increase dose, check in 1 week |
| 7/2/24 | 4 mg BID | 81 ng/mL | none | Reviewed risks of low rivaroxaban level with mom, mom opted to continue current dose and check in 1 month |
| 7/30/24 | 4 mg BID | 162 ng/mL | none | Check in 2 months |
| 9/26/24 | 4 mg BID | 74 ng/mL | none | Reviewed options to continue current dose, increase dose, or change to enoxaparin. Mom opted to continue current dose but change administration to oral instead of by GT, check in 1 month |
| 10/22/24 | 4 mg BID | 202 ng/mL | none | Check in 1 month |
| 11/14/24 | 4 mg BID | 89 ng/mL | none | Check in 1 month |
| 12/16/24 | 4 mg BID | 96 ng/mL | none | Check in 1 month |

Supplemental Table 1c. Dosage changes with corresponding levels and comments for Case 3
