# Supplementary material for: Rivaroxaban as Secondary Thromboprophylaxis in Pediatric Patients With Short Bowel Syndrome
Source: Case Rep Hematol. 2026 Sep 29;2026:7383741. doi: 10.1155/crh/7383741 (PMC13621405; doi:10.1155/crh/7383741)
Supplement: Supplementary file 2 [file CRH-2026-7383741-s002.docx]

**CARE Checklist**

|  |  | Reporting Item | Page# Line # |
| --- | --- | --- | --- |
| **Title** |  |  |  |
|  | [#1](https://www.goodreports.org/reporting-checklists/care/info/#1) | The area of focus and “case report” should appear in the title | **1; 1,2** |
| **Keywords** |  |  |  |
|  | [#2](https://www.goodreports.org/reporting-checklists/care/info/#2) | Two to five key words that identify topics in this case report | **1; 33** |
| **Abstract** |  |  |  |
| Introduction | [#3a](https://www.goodreports.org/reporting-checklists/care/info/#3a) | What is unique and why is it important? | **1; 17-19** |
|  | [#3b](https://www.goodreports.org/reporting-checklists/care/info/#3b) | The patient’s main concerns and important clinical findings. | **1; 17-20** |
|  | [#3c](https://www.goodreports.org/reporting-checklists/care/info/#3c) | The main diagnoses, interventions, and outcomes. | **1; 21-23** |
| Conclusion | [#3d](https://www.goodreports.org/reporting-checklists/care/info/#3d) | What are one or more “take-away” lessons? | **1; 23-25** |
| **Introduction** |  |  |  |
|  | [#4](https://www.goodreports.org/reporting-checklists/care/info/#4) | Briefly summarize why this case is unique with medical literature references. | **1, 2; 36-61** |
| **Patient information** |  |  |  |
|  | [#5a](https://www.goodreports.org/reporting-checklists/care/info/#5a) | De-identified demographic and other patient information. | **2, 3; 83, 100, 120** |
|  | [#5b](https://www.goodreports.org/reporting-checklists/care/info/#5b) | Main concerns and symptoms of the patient. | **2, 3; 83-84 100-101, 120-121** |
|  | [#5c](https://www.goodreports.org/reporting-checklists/care/info/#5c) | Medical, family, and psychosocial history including genetic information. | **N/A** |
|  | [#5d](https://www.goodreports.org/reporting-checklists/care/info/#5d) | Relevant past interventions and their outcomes. | **N/A** |
| **Clinical findings** |  |  |  |
|  | [#6](https://www.goodreports.org/reporting-checklists/care/info/#6) | Relevant physical examination (PE) and other clinical findings. | **Table 1** |
| **Timeline** |  |  |  |
|  | [#7](https://www.goodreports.org/reporting-checklists/care/info/#7) | Relevant data from this episode of care organized as a timeline (figure or table). | **Figure 1 & Supplemental Tables 1a, 1b, and 1c** |
| **Diagnostic assessment** |  |  |  |
|  | [#8a](https://www.goodreports.org/reporting-checklists/care/info/#8a) | Diagnostic methods (PE, laboratory testing, imaging, surveys). | **2; 67-75** |
|  | [#8b](https://www.goodreports.org/reporting-checklists/care/info/#8b) | Diagnostic challenges. | **N/A** |
|  | [#8c](https://www.goodreports.org/reporting-checklists/care/info/#8c) | Diagnostic reasoning including differential diagnosis | **N/A** |
|  | [#8d](https://www.goodreports.org/reporting-checklists/care/info/#8d) | Prognostic characteristics when applicable | **N/A** |
| **Therapeutic Intervention** |  |  |  |
|  | [#9a](https://www.goodreports.org/reporting-checklists/care/info/#9a) | Types of intervention (pharmacologic, surgical, preventive). | **Supplemental Tables 1a, 1b, and 1c** |
|  | [#9b](https://www.goodreports.org/reporting-checklists/care/info/#9b) | Administration of intervention (dosage, strength, duration) | **Supplemental Tables 1a, 1b, and 1c** |
|  | [#9c](https://www.goodreports.org/reporting-checklists/care/info/#9c) | Changes in the interventions with explanations. | **Supplemental Tables 1a, 1b, and 1c** |
| **Follow up and outcomes** |  |  |  |
|  | [#10a](https://www.goodreports.org/reporting-checklists/care/info/#10a) | Clinician and patient-assessed outcomes when appropriate | **N/A** |
|  | [#10b](https://www.goodreports.org/reporting-checklists/care/info/#10b) | Important follow-up diagnostic and other test results. | **2, 3; 92-98, 113-119, 132-137** |
|  | [#10c](https://www.goodreports.org/reporting-checklists/care/info/#10c) | Intervention adherence and tolerability (how was this assessed)? |  |
|  | [#10d](https://www.goodreports.org/reporting-checklists/care/info/#10d) | Adverse and unanticipated events. | **2, 3; 115-118, 132-137** |
| **Discussion** |  |  |  |
|  | [#11a](https://www.goodreports.org/reporting-checklists/care/info/#11a) | Strengths and limitations in your approach to this case. | **4,5; 155-209** |
|  | [#11b](https://www.goodreports.org/reporting-checklists/care/info/#11b) | Discussion of the relevant medical literature. | **4,5; 140-209** |
|  | [#11c](https://www.goodreports.org/reporting-checklists/care/info/#11c) | The rationale for your conclusions. | **4,5; 155-209** |
|  | [#11d](https://www.goodreports.org/reporting-checklists/care/info/#11d) | The primary “take-away” lessons from this case report. | **5; 206-209** |
| **Patient perspective** |  |  |  |
|  | [#12](https://www.goodreports.org/reporting-checklists/care/info/#12) | The patient can share their perspective on their case | **N/A** |
| **Informed consent** |  |  |  |
|  | [#13](https://www.goodreports.org/reporting-checklists/care/info/#13) | The patient should give informed consent. | **This study was approved by the Institution Review Boards at the University of Utah and Primary Children’s Hospital, Salt Lake City, UT, United States (IRB_00188583). Consent was waived due to the retrospective nature of the study and approved under the institutional IRB.** |
